# Prostate Cancer Risk Calculators for Healthy Populations: Systematic Review

**DOI:** 10.2196/30430

**Published:** 2021-09-03

**Authors:** Antonio Bandala-Jacques, Kevin Daniel Castellanos Esquivel, Fernanda Pérez-Hurtado, Cristobal Hernández-Silva, Nancy Reynoso-Noverón

**Affiliations:** 1 Centro de Investigación en Prevención Instituto Nacional de Cancerología Mexico City Mexico; 2 Centro de Investigación en Salud Poblacional Instituto Nacional de Salud Pública Mexico City Mexico

**Keywords:** prostate cancer, risk calculator, risk reduction

## Abstract

**Background:**

Screening for prostate cancer has long been a debated, complex topic. The use of risk calculators for prostate cancer is recommended for determining patients’ individual risk of cancer and the subsequent need for a prostate biopsy. These tools could lead to better discrimination of patients in need of invasive diagnostic procedures and optimized allocation of health care resources

**Objective:**

The goal of the research was to systematically review available literature on the performance of current prostate cancer risk calculators in healthy populations by comparing the relative impact of individual items on different cohorts and on the models’ overall performance.

**Methods:**

We performed a systematic review of available prostate cancer risk calculators targeted at healthy populations. We included studies published from January 2000 to March 2021 in English, Spanish, French, Portuguese, or German. Two reviewers independently decided for or against inclusion based on abstracts. A third reviewer intervened in case of disagreements. From the selected titles, we extracted information regarding the purpose of the manuscript, analyzed calculators, population for which it was calibrated, included risk factors, and the model’s overall accuracy.

**Results:**

We included a total of 18 calculators from 53 different manuscripts. The most commonly analyzed ones were the Prostate Cancer Prevention Trial (PCPT) and European Randomized Study on Prostate Cancer (ERSPC) risk calculators developed from North American and European cohorts, respectively. Both calculators provided high diagnostic ability of aggressive prostate cancer (AUC as high as 0.798 for PCPT and 0.91 for ERSPC). We found 9 calculators developed from scratch for specific populations that reached a diagnostic ability as high as 0.938. The most commonly included risk factors in the calculators were age, prostate specific antigen levels, and digital rectal examination findings. Additional calculators included race and detailed personal and family history.

**Conclusions:**

Both the PCPR and ERSPC risk calculators have been successfully adapted for cohorts other than the ones they were originally created for with no loss of diagnostic ability. Furthermore, designing calculators from scratch considering each population’s sociocultural differences has resulted in risk tools that can be well adapted to be valid in more patients. The best risk calculator for prostate cancer will be that which has been calibrated for its intended population and can be easily reproduced and implemented.

**Trial Registration:**

PROSPERO CRD42021242110; https://www.crd.york.ac.uk/prospero/display_record.php?RecordID=242110

## Introduction

According to the World Health Organization, the 2020 global incidence of prostate cancer was 1,414,259 cases, which represented 7.3% of all the new cancer cases. It represents the fourth most common type of cancer [1]. In Mexico, prostate cancer is the leading type of cancer death in men 50 years and older [2]. Early prostate cancer detection could help to accurately discriminate indolent from aggressive cancers and significantly reduce the overuse of invasive diagnostic techniques and the side effects associated with cancer treatment [3]. A randomized study on the European population who underwent screening showed a progressive 51% reduction in prostate cancer mortality in individuals up to age 75 years [4].

Currently, there is no evidence to support or refute the implementation of widespread early screening programs for prostate cancer; and the position of international guidelines on who and when to screen has constantly pivoted. Thus, active surveillance must be based carefully on individualized weight of risk factors [5,6]. For example, the combination of family history of prostate cancer, personal medical history, serum biomarker levels, and sociocultural aspects has led to the creation of tools that can more accurately predict individual risk for prostate cancer and focalize screening strategies for populations at higher risk. These tools, or risk calculators, could lead to a reduction in the overdiagnosis of prostate cancer and its subsequent overtreatment [7]. The European Randomized Study of Prostate Cancer (ERSPC) risk calculator (RC) and the Prostate Cancer Prevention Trial (PCPT) RC are two well-known prostate cancer risk calculators that have been extensively validated in independent cohorts in their original versions; recent, updated versions of both calculators have shown promising results in populations other than the ones for which they were originally developed [8]. Other well-known, externally validated predictive models like the Prostate Health Index (PHI), which includes more biomarkers, are important tools in reducing unnecessary prostate biopsies [9]. All of these predictive models have been used among diverse populations with different results regarding each risk factor’s individual predictive value for prostate cancer, as well as the models’ overall performance.

Prostate cancer screening is based in the combination of serum prostate specific antigen (PSA), digital rectal examination (DRE), and sometimes additional urine biomarkers. Additional tools such as magnetic resonance imaging (MRI) and risk calculators may help decide the need for a biopsy [10]. Advanced imaging techniques and access to biopsy are not always available, particularly in lower- and middle-income countries, which renders risk calculators a viable option to decide which patients are in need of additional screening and for optimizing allocation of health care resources. A systematic review on prostate cancer risk calculators in a healthy population could summarize current tools available to primary care physicians and encourage the adaptation or creation of new risk calculators adjusted to each population’s sociocultural variations [11].

The aim of our study was to systematically review available literature on current prostate cancer risk calculators in healthy population by comparing the relative impact of individual items on different cohorts and the models’ overall performance.

## Methods

### Search Methods

A systematic review was performed in April 2021. We searched MEDLINE via PubMed and Latin American and Caribbean Health Sciences via LILACS for publications between January 1, 2000 and April 1, 2021. We used 3 combined queries as follows: (“2000/01/01”[Date–Publication]: “2021/04/01”[Date–Publication]) AND ((cancer of prostate [MeSH terms]) OR (prostate cancer [MeSH terms])) OR (prostate cancer [text word]) AND ((risk prediction [text word]) OR (risk model [text word])) OR (risk calculator [text word]). We extracted the resulting titles and abstracts into a spreadsheet. This systematic review was registered at PROSPERO [CRD42021242110].

### Selection Criteria

Articles were included if they met the following criteria:

- Authors presented a new risk calculator for prostate cancer OR authors validated or modified an existing risk calculator in a different population OR authors compared predictive capabilities of 2 or more risk calculators
- Article was in either Spanish, English, French, Portuguese, or German
- Article explicitly described the calculator’s predictive capability

Articles were excluded if any of the following were true:

- Article presented or analyzed a calculator for nonhealthy population such as models to predict aggressiveness or relapse in a population already diagnosed with prostate cancer
- Reported risk factors were mainly genomic (eg, polymorphisms) or considered inaccessible for general practitioners or in settings with limited resourced (eg, MRI)

### Data Extraction and Analysis

Using the listed criteria, two authors independently reviewed the titles and abstracts and decided for or against inclusion. We included titles if both reviewers agreed on inclusion and vice versa for exclusion. If the reviewers disagreed, a third reviewer decided on the article’s inclusion. We then obtained the full text for selected titles, screened them for final inclusion eligibility, and extracted the data from selected articles. From each included article, we extracted the objective, study design, number of participants and their inclusion criteria, name of the proposed or analyzed model, methodology for the development or analysis of each model’s included risk factors and their impact measurements, validation methodology, and each model’s prediction capability. From the extracted data, we then summarized the risk factors and their impact measurements for prostate cancer according to each model that included them.

## Results

**Figure 1:**
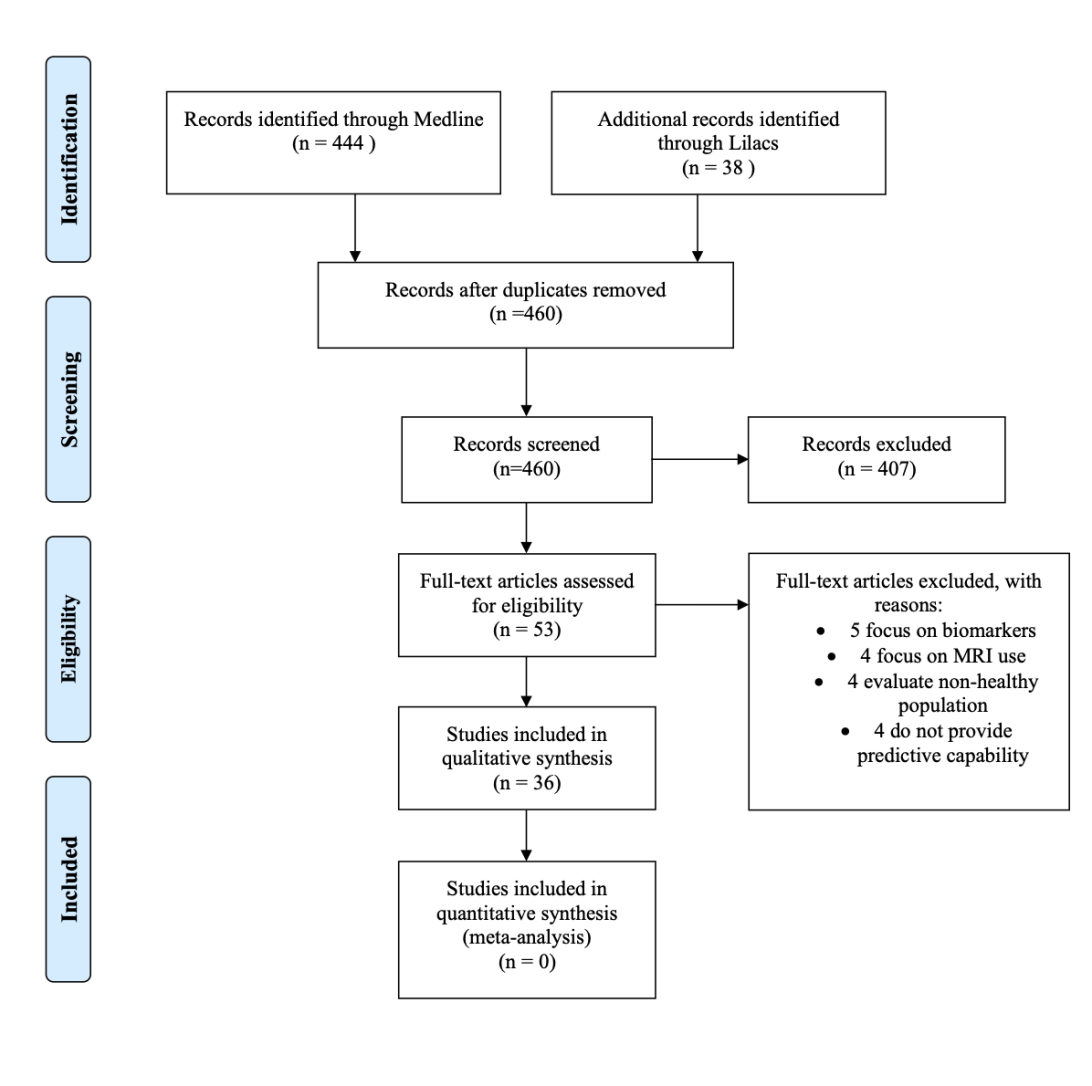
PRISMA flowchart of included studies.

Our search resulted in 460 articles after excluding duplicates. We reviewed all results and agreed on 53 articles that passed the title and abstract stage, in which we evaluated the complete text. We then excluded an additional 17 titles: 5 that focused on biomarkers as predictors, 4 that evaluated the use of MRI techniques, 4 on nonhealthy population that predicted recurrence of disease, and 4 that did not specify risk or prediction ability. We then extracted information on the remaining 36 studies and classified them as articles that evaluated or calibrated risk calculators in a new population, studies that compared 2 or more existing risk calculators in a specific population, and studies that proposed and validated a novel risk calculator. We identified a total of 18 risk calculators in the 36 included studies. We did not perform a metanalysis of the individual risk factors as the reported impact measurements were too heterogenous (Figure 1).

We first identified the most commonly studied risk calculators and the risk factors they include in their original versions. The most mentioned risk calculators were the ERSPC, PCPT, and PHI.

The ERSPC RC, in its original version for use by medical personnel (R3 version), includes MRI information if available, PSA levels, results of a prior biopsy, and results of a DRE and prostate volume measured by transrectal ultrasound [12]. In its original version, the PCPT RC includes age, race, PSA levels, family history of prostate cancer, results of a DRE, results of a prior biopsy, and when available, free PSA, prostate cancer antigen 3, and T2:ERG [13]. On the other hand, the PHI calculates risk with a mathematical approach that includes PSA, free PSA, and prostate specific antigen isoform p2 [14]. Since their introduction, all these calculators have undergone external modifications with additional risk factors. Additionally, we found calculators that were developed de novo and that include different risk factors from the canonical ERPSC and PCPT RCs. For example, the Lifestyle Risk Prediction Model for Prostate Cancer by Kim et al [15] includes height, weight, glucose levels, meat and alcohol consumption, smoking status, and physical activity. The risk calculator by Albright et al [16] incorporates a detailed extended family history to calculate the risk of prostate cancer, and the risk calculator by Jalali et al [17] combines traditional measurements of PSA and DRE with family history.

In Table 1, we present the summary of all articles in the systematic review. A single article may have evaluated multiple risk calculators or may have had multiple purposes. A total of 18 articles evaluated the PCPT RC (1 optimized it with the prostate health index, 1 optimized it with detailed family history, 14 calibrated or assessed it in a new population, and 2 assessed it in a new population while also comparing it with a different calculator). Similarly, 14 articles evaluated the ERSPC RC in its level 3 version (1 optimized it with the PHI, and 13 calibrated it in a new population, out of which 7 also compared it to a different calculator [essentially the PCPT RC or a new calculator]). We found 9 articles describing a new risk calculator as well as their area under the curve (AUC) and calibration. The table also describes the predictive capacity that each study found for the analyzed risk calculators. For example, depending on the populations in which they were used, the PCPT RC had AUCs ranging from as low as 0.562 to as high as 0.813, while the ERSPC RC reported AUCs from 0.68 to 0.86. These AUCs also varied depending on whether the calculator was applied to any prostate cancer or to high-grade prostate cancer. AUCs are generally higher when looking for high-grade cancers. For example, for the PCPT RC, AUCs for prostate cancer peaked at 0.783, while those for high-grade prostate cancer could be as high as 0.813. Furthermore, risk calculators created from scratch also showed high predictive capabilities on their target population, such as the Korean Prostate Cancer Risk Calculator (AUC 0.887) by Kim et al [15], which uses socioenvironmental aspects of their population to create the predictive models, and the risk calculator by Albright et al [16], which stratifies risk depending on the number of extended family members with prostate cancer.

In Table 2, we detail the reported impact measures associated with each risk factor by risk calculator. Not all studies specified the impact measures of each individual risk factor but rather reported only the calculator’s overall predictive ability, as described in Table 2. For those that did specify, elevated PSA levels and a positive DRE conferred the highest risk for prostate cancer. For example, log PSA as a predictor in the PCPT RC conferred an HR of 5.42 and an OR of 1.8 for prostate cancer, while a positive DRE showed significant ORs from 2.2 to as high as 8.22 in the Korean Prostate Cancer RC. A positive family history of prostate cancer also conferred higher odds in the PCPT RC. On the other hand, and as is expected, a prior negative biopsy was found as a protective factor for prostate cancer (with HRs of 0.14 and 0.64 as found in the PCPT RC). Race was not significant in any of the calculators that specified its impact measures.

**Table 1:** Summary of models in included studies.

Model and country			Article		Year		Purpose		End point		Sample size		Study type		AUC^a^		Notes
**PCPT^b^RC^c^**																	
	Ireland	Loeb, et al [18]		2017		A^d^		Gleason ≥7		892		1^e^		0.697		Inclusion of PHI^f^ into an existing calculator	
	US	Auffenberg, et al [19]		2017		B^g^		Absence of cancer, Gleason <7, Gleason ≥7		11,809		2^h^		0.621 (0.607-0.64)		—^i^	
	Ireland	Lundon, et al [20]		2015		B		Risk of any prostate cancer diagnosis and risk of high-grade disease		556		2		PC^j^: 0.628; high-grade PC: 0.798		—	
	Switzerland	Poyet, et al [21]		2016		B		Gleason ≥7 and/or T stage ≥T2b		1615		2		PC: 0.66; high-grade PC: 0.69		Validation in a Swiss cohort	
	North America and Europe	Ankerst, et al [22]		2018		B,C^k^		Gleason ≥7, <7, or no cancer		15,611		2		0.723 (0.709-0.737)		Compares AUC to PBCG^l^ RC	
	Switzerland	Poyet, et al [11]		2016		B		PC or significant PC (Gleason ≥7)		1996		2		PC: 0.66; significant PC: 0.70		Compares and calibrates new versions of PCPT and ERSPC^m^	
	US	Kaplan, et al [23]		2010		B		PC or significant PC (Gleason ≥7)		624		1		Not specified		Validates PCPT in high-risk individuals	
	International	Ankerst, et al [13]		2012		B		Each cohort’s criteria		25,733		2		ERSPC Goeteborg 1: 0.72; ERSPC Goeteborg 2-6: 0.562; ERSPC Rotterdam 1: 0.7; ERSPC Rotterdam 2-3: 0.61; ERSPC Tarn: 0.667; SABOR^n^: 0.654; Cleveland Clinic: 0.588; ProtecT: 0.639; Tyrol: 0.667; Durham: 0.715		—	
	Portugal	Cavadas, et al [24]		2010		B,C		Positive biopsy		493		2		0.744 (0.705-0.781)		—	
	Sweden	Grill, et al [25]		2015		A		Same as PCPT RC		55,158 cases + 632,218 controls		3^o^		Not specified		Adds detailed family history to PCPT RC	
	Canada	Trottier, el al [26]		2011		B,C		PC or high-grade PC		982		2		0.63		—	
	US	Carbunaru, et al [27]		2019		B,C		PC and significant PC		954		2		Significant PC: 0.64 (0.61-0.68)		—	
	Mexico	Liang, et al [28]		2013		B		PC and significant PC		826		2		PC: 0.785; high-grade PC: 0.766		—	
	US	Nguyen, et al [29]		2010		B		PC or high-grade PC		3482		2		PC: 0.57; high-grade PC: 0.6		—	
	China	Zhu, et al [30]		2012		B,C		PC or high-grade PC		495		2		PC: 0.783 (0.737-0.83); high-grade PC: 0.813 (0.764-0.862)		—	
	US	Liang, et al [31]		2013		B		PC or high-grade PC		1021		2		Not specified		—	
	US	Nam, et al [32]		2011		B,C		PC or high-grade PC		2130		2		PC: 0.61 (0.59-0.64); aggressive PC: 0.67 (0.64-0.7)		—	
	US	Parekh, et al [14]		2006		B		PC or high-grade PC		446		2		0.655 (0.602-0.708)		Uses PCPT in an ethnically diverse population	
**Finasteride-adjusted PCPT RC**																	
	Mexico	Liang, et al [33]		2012		B		PC		837		2		PC: 0.784; high-grade PC: 0.768		—	
**ERSPC RC (level 3)**																	
	US	Loeb, et al [18]		2017		A		Gleason ≥7		892		1		0.711		Inclusion of PHI into an existing calculator	
	Europe	Van Vugt, et al [34]		2010		B		Positive sextant prostate biopsy		1825 Finnish men + 531 Swedish men		2		Finnish cohort 0.76 (0.74-0.79), Swedish cohort 0.78 (0.73-0.83)		—	
	Netherlands	Gayet, et al [35]		2018		B		Gleason ≥7 and/or T stage ≥T2b		1812		2		PC: 0.78 (0.76-0.8); significant PC: 0.91 (0.89-0.92)		—	
	Ireland	Lundon, et al [20]		2015		B,C		Risk of any PC diagnosis and risk of high-grade disease		556		2		PC: 0.588; high-grade PC: 0.69		—	
	South Africa	Kowlessur, et al [36]		2020		B		Gleason ≥7 and/or T stage ≥T2b		475		2		PC: 0.738 (0.695-0.781); significant PC: 0.833 (0.789-0.876)		Calibration of ERSPC for South African Population	
	Switzerland	Poyet, et al [21]		2016		B		Gleason ≥7 and/or T stage ≥T2b		1615		2		PC: 0.64; high-grade PC: 0.70		Validation in a Swiss cohort	
	Spain	Gómez-Gómez, et al [37]		2017		B,D^p^		Gleason ≥7 and/or T stage ≥T2b		749		2		PC: 0.69 (0.65-0.74), high-grade PC: 0.74 (0.70-0.79)		Also evaluates variability with a subsequent PSA^q^ sample	
	Switzerland	Poyet, et al [11]		2016		B,C		PC or high-grade PC		1996		2		PC: 0.65; significant PC: 0.73		Compares and calibrates new versions of PCPT and ERSPC	
	Canada	Trottier, et al [26]		2011		B,C		PC or high-grade PC		982		2		0.71		—	
	China, Netherlands	Chen, et al [38]		2021		B,C		PC or high-grade PC		6741		2		European cohort: PC: 0.79 (0.77-0.81); high-grade PC: 0.86 (0.84-0.89); Chinese cohort: PC: 0.74 (0.72-0.76); high-grade PC: 0.74 (0.72-0.76)		Compares CPCC^r^ RC to ERSPC RC	
	Portugal	Cavadas, et al [24]		2010		B,C		Positive biopsy		493		2		0.801 (0.764-0.834)		—	
	Netherlands	Van Vugt, et al [39]		2012		B		Positive sextant prostate biopsy		320		2		0.77 (0.72-0.83)		—	
	China	Zhu, et al [30]		2012		B,C		PC or high-grade PC		495		2		PC: 0.831 (0.79-0.872); high-grade PC: 0.852 (0.807-0.897)		—	
	Europe	Roobol, et al [40]		2015		B,C,D		Positive sextant prostate biopsy		1185		2		PC: 0.72; clinically relevant PC: 0.68		Uses an ERSPC model that includes PHI	
**ERSPC RC (level 4)**																	
	Netherlands	Gayet, et al [35]		2018		B		Gleason ≥7 and/or T stage ≥T2b		1812		2		PC: 0.62 (0.56-0.67); significant PC: 0.74 (0.66-0.81)		—	
	Europe	Roobol, et al [40]		2015		B,C,D		Positive sextant prostate biopsy		1185		2		PC: 0.72 (0.67-0.77)		Uses an ERSPC model that includes PHI	
**MUSIC** ^s^ **model**																	
	US	Auffenberg, et al [19]		2017		A,C		Absence of cancer, Gleason <7, Gleason ≥7		11,809		2		0.63 (0.613-0.65)		—	
**CPCC RC**																	
	China	Chen, et al [41]		2016		A,C		PC or high-grade PC		924 patients for model development + 911 patients for model validation		2		PC: 0.801 (0.771-0.831); high-grade PC: 0.826 (0.796-0.857)		Compares CPCC RC to ERSPC RC	
	China, Netherlands	Chen, et al [38]		2021		B,C		PC or high-grade PC		6741		2		European cohort: PC: 0.77 (0.75-0.79); high-grade PC: 0.86 (0.83-0.88); Chinese cohort: PC 0.77 (0.74-0.77); high-grade PC: 0.77 (0.75-0.79)		—	
**ProstateCheck**																	
	Switzerland	Poyet, et al [21]		2016		B,C		Gleason ≥7 and/or T stage ≥T2b		1615		2		PC: 0.69 (0.67-0.73); high-grade PC: 0.72 (0.69-0.77)		ProstateCheck is based on the ERSPC	
**Sunnybrook normogram-based PC RC**																	
	US	Nam, et al [32]		2011		B,C		PC or high-grade PC		2130		2		PC: 0.67 (0.65-0.69); aggressive PC: 0.72 (0.7-0.75)		—	
**PHI model**																	
	Ireland	Foley, et al [42]		2016		D		Low grade PCA: Gleason 6; High-grade PCA: Gleason ≥7.		250		2		PC: 0.71; high-grade PC: 0.78		Development of a model that incorporates PHI score	
**PBCG RC**																	
	North America and Europe	Ankerst, et al [22]		2018		D,C		Gleason ≥7, <7, or no cancer		15,611		2		0.755 (0.742-0.768)		Compares AUC to PCPT RC	
	US	Carbunaru, et al [27]		2019		B,C		PC and significant PC		954		2		Significant PC: 0.65 (0.62-0.68)			
**Next-generation PC RC**																	
	Canada	Nam, et al [43]		2018		D		Gleason ≥7		5639 patients with a prostate biopsy + 979 patients with PC		2		Model 1: concordance index 0.74 (0.72-0.76); model 2: concordance index 0.71 (0.69-0.72)		—	
**Seoul National University PC RC**																	
	South Korea	Jeong, et al [44]		2014		D,C		PC		3482		2		Development cohort: 0.786; validation cohort: 0.811		Mobile app-based RC	
**Indonesian PC RC**																	
	Indonesia	Yuri, et al [45]		2015		D,C		Not specified		1957		2		0.938 (0.93-0.95)		—	
**Korean PC RC**																	
	South Korea	Yoon, et al [46]		2012		D		Positive biopsy		602		2		0.9 (0.89-0.92)		—	
**Unnamed model by Albright, et al**																	
	US	Albright, et al [16]		2015		D		PC		635,433		2		Not specified		Model uses extended detailed family history	
**Unnamed model by Loeb, et al**																	
	US	Loeb, et al [18]		2017		D		Gleason ≥7		892		1		0.746		Development of a model that incorporates PHI score	
**Unnamed model by Kim, et al**																	
	South Korea	Kim, et al [15]		2018		D		ICD-10 code C61		1,179,172 for model development + 389,539 for model validation		2		0.887 (0.879-0.895)		Based on epidemiologic factors rather than PSA	
**Unnamed model by Jalali, et al**																	
	Ireland	Jalali, et al [17]		2020		D,C		PC or high-grade PC		4801		2		PC: 0.674 (0.659-0.689); high-grade PC: 0.721 (0.701-0.741)		Calculator informs need for prostate biopsy	
**Unnamed model by Chen, et al**																	
	Taiwan	Chen, et al [47]		2020		A,D		PC or high-grade PC		1545		2		PC: 0.795; high-grade PC: 0.869		App-based calculator	

^a^AUC: area under the curve.

^b^PCPT: Prostate Cancer Prevention Trial.

^c^RC: risk calculator.

^d^A: optimizes an existing model.

^e^1: clinical trial.

^f^PHI: prostate health index.

^g^B: calibrates and/or assesses discrimination of an existing model in a specific population.

^h^2: cohort.

^i^PC: prostate cancer.

^j^C: compares two or more existing models in a specific population.

^k^PBCG: Prostate Biopsy Collaborative Group.

^l^ERSPC: European Randomized Study on Screening for Prostate Cancer.

^m^SABOR: San Antonio Center of Biomarkers of Risk for Prostate Cancer.

^n^3: case control.

^o^D: presents and validates a new model.

^p^PSA: prostate specific antigen.

^q^CPCC: Chinese Prostate Cancer Consortium.

^r^MUSIC: Michigan Urological Surgery Improvement Collaborative.

**Table 2:** Impact measure of risk factors included in prostate cancer risk calculators.

Risk factor and model		Author	Impact measure	*P* value	Notes
**Age**					
	ERSPC^a^ RC^b^	Trottier, et al [26]	Mean risk 0.31	—^c^	Age >70
	PCPT^d^ RC	Trottier, et al [26]	Mean risk 0.53	—	Age >70
	CPCC^e^ RC model 1	Chen, et al [41]	OR^f^ 1.074 (1.050-1.098)	<.001	—
	Unnamed model by Kim, et al	Kim, et al [15]	HR^g^ 1.26 (1.245-1.276)	<.001	As “age-mean_age”
	Korean PC^h^ RC	Yoon, et al [46]	OR 1.06 (1.04-1.08)	<.001	—
**Race**					
	ERSPC RC	Trottier, et al [26]	Mean risk 0.25	—	Hispanic
	PCPT RC	Kaplan, et al [23]	HR 1.1 (0.58-2.08)	.76	African American race
	—	Trottier, et al [26]	Mean risk 0.48	—	Hispanic
**Family history of PC**					
	ERSPC RC	Trottier, et al [26]	Mean risk 0.28	—	—
	PCPT RC	Kaplan, et al [23]	HR 1.16 (0.60-2.25)	.67	—
	—	Trottier, et al [26]	Mean risk 0.51	—	—
	—	Yuang, et al [28]	OR 1.31 (1.11-1.55)	<.001	—
	Unnamed model by Liang Y, et al	Yuang, et al [28]	OR 3.23 (1.89-5.54)	<.001	—
**PSA** ^i^					
	ERSPC RC	Trottier, et al [26]	Mean risk 0.35	—	>6 ng/mL
	PCPT RC	Kaplan, et al [23]	HR 5.42 (3.90-7.52)	—	As log PSA
	—	Trottier, et al [26]	Mean risk 0.56	—	>6 ng/mL
	—	Yuang, et al [28]	OR 1.8 (1.46-2.21)	<.001	As log PSA
	CPCC RC model 1	Chen, et al [41]	OR 7.7219 (4.3644-13.6625)	<.001	As log PSA
	Korean PC RC	Yoon, et al [46]	OR 4.31 (3.29-5.65)	<.001	As log PSA
	Unnamed model by Liang Y, et al	Yuang, et al [28]	OR 2.34 (2.13-2.56)	<.001	As log PSA
**Free PSA**					
	CPCC RC model 1	Chen, et al [41]	OR 0.015 (0.0016-0.1407)	<.001	As free PSA ratio
	Korean PC RC	Yoon, et al [46]	OR 2.74 (2.12-3.40)	<.001	As log free PSA
**DRE [+]** ^j^					
	ERSPC RC	Trottier, et al [26]	Mean risk 0.45	—	—
	PCPT RC	Kaplan, et al [23]	HR 0.45 (0.16-1.24)	.12	—
	—	Trottier, et al [26]	Mean risk 0.61	—	—
	—	Yuang, et al [28]	OR 2.47 (2.03-3.01)	<.001	—
	CPCC RC model 1	Chen, et al [41]	OR 2.2031 (1.5268-3.1788)	<.001	—
	Unnamed model by Liang Y, et al	Yuang, et al [28]	OR 4.22 (2.91-6.14)	<.001	—
	Korean PC RC	Yoon, et al [46]	OR 8.22 (5.44-12.4)	<.001	—
**Previous biopsy**					
	ERSPC RC	Trottier, et al [26]	Mean risk 0.15	—	—
	PCPT RC	Kaplan, et al [23]	HR 0.14 (0.05-0.37)	<.001	Prior negative biopsy
	—	Trottier, et al [26]	Mean risk 0.45	—	—
	—	Yuang, et al [28]	OR 0.64 (0.53-0.78)	<.001	Prior negative biopsy
	Unnamed model by Liang Y, et al	Yuang, et al [28]	OR 0.13 (0.07-0.23)	<.001	Prior negative biopsy
**TRU** ^k^					
	ERSPC RC	Trottier, et al [26]	Mean risk 0.2	—	≥42 mL
	PCPT RC	Trottier, et al [26]	Mean risk 0.49	—	≥42 mL
	Korean PC RC	Yoon, et al [46]	OR 4.05 (2.79-5.88)	—	—

^a^ERSPC: European Randomized Study on Screening for Prostate Cancer.

^b^RC: risk calculator.

^c^Not applicable.

^d^PCPT: Prostate Cancer Prevention Trial.

^e^CPCC: Chinese Prostate Cancer Consortium.

^f^OR: odds ratio.

^g^HR: hazard ratio.

^h^PC: prostate cancer.

^i^PSA: prostate specific antigen.

^j^DRE [+]: positive/altered digital rectal examination.

^k^TRU: transrectal ultrasound.

## Discussion

### Principal Findings

Our study’s most important findings were that most available risk prediction tools for prostate cancer are optimizations (ie, improvement of the predictive capacity of existing calculators) or recalibration (ie, applying an existing one to a different population) of the PCPT RC or ERSPC RC. Furthermore, some authors presented and validated a new calculator from scratch. Whatever the mechanism, all risk calculators that have been optimized, calibrated, or created with a specific population in mind seem to have adequately high prediction capabilities.

In our study, we have provided a comprehensive description of available risk calculators for prostate cancer and their predictive capability in healthy population. Due to the nature of prostate cancer; when, who, and even if, to screen, has always been a controversial topic. Before the PSA era, overdiagnosis and overtreatment were major concerns. Since the implementation of PSA screening, there has been a reported decrease of 53% in prostate cancer mortality in the United States. However, North American guidelines have shifted between their position to screen or not using PSA [48]. Furthermore, the recent introduction of novel serum-based models that complement PSA, such as the PHI, have improved the detection capability of clinically significant prostate cancer. A combination of several individual factors into a prediction model could more accurately predict cases of prostate cancer that need to be treated and reduce the number of unnecessary biopsies and their complications [49].

Although there have been recent improvements in detection of prostate cancer with the use of novel biomarkers and advanced imaging techniques, these are not widely available, especially in low- and middle-resource settings, and cannot be widely applicable at the primary level, which renders the use of reproducible predictive models based on data available at primary settings essential for decision making at a larger scale. Despite this, the two most commonly used models for predicting prostate cancer, the PCPT RC and ERSPC RC, were created and validated with North American and European populations and may not have the same predictive capabilities when applied as they are, in different populations. To further emphasize this, people of non-European ancestries make up less than 15% of the available genome-wide association study of prostate cancer [50]. However, our systematic review found numerous cases of calibration of these tools for different population with results similar to the originals. One of such examples is the external validation by Chen et al [47] of the ERSPC RC in a Chinese cohort, in which they found an AUC of 0.74 for any prostate cancer and a similar AUC of 0.74 for high-grade prostate cancer, while also finding in the same cohort an AUC of 0.77 for any or high-grade prostate cancer using the Chinese Prostate Cancer Consortium (CPCC) RC. They thus concluded that an Asian-adapted ERSPC RC and application of the CPCC RC in a European PSA-based screening reduce unnecessary biopsies; however, they stress the need for external validation before implementing a risk calculator.

Still, our review found that fewer than 10 of the included articles focused on calibrating these calculators on non-European or non–North American populations: most of them in Asia, 1 in South Africa, and 1 in Mexico. The underrepresentation of an ethnically diverse population for the calibration of these tools results in fewer available predictive models in the settings where they would be most beneficial. For example, the study by Liang et al [28] of the PCPT RC in a Mexican population resulted in an AUC of 0.785 for high-grade prostate cancer, even higher than the tool’s AUC when applied to European populations in other studies. Similarly, the calibration by Kowlessur et al [36] of the ERSPC RC for a South African population resulted in a high AUC of 0.833 for high-grade prostate cancer. Knowing that these tools can be easily adapted and calibrated for populations in lower-resource settings could encourage researchers to adjust these calculators to settings that still struggle with the overperformance of invasive biopsies.

Although the characteristics of the included studies did not allow for a meta-analysis of the individual risk factors or the tools’ overall predictive capabilities, it seems that both the ERSPC RC and PCPT RC have similarly high predictive capabilities. Zhu et al [30] reported an AUC of up to 0.813 for the PCPT RC in a Chinese cohort, and Gayet et al [35] reported an AUC of 0.91 in the ERSPC RC in a Dutch cohort. Either of these calculators could be potentially adapted to new populations depending on the availability of transrectal ultrasound, which is one of the included items for calculating risk in the ERSPC RC that the PCPT RC does not include. In the end, it is not about determining which risk calculator is best but about making sure that whichever one is used is calibrated and adapted to its intended recipients. That is, the best calculator will be one that is accessible, valid, and reproducible.

The creation of new tools targeted at new populations is also a valid alternative to calibrating existing ones, and this can also yield optimal results. For example, the calculator by Yuri et al [45] designed for an Indonesian population resulted in an AUC of 0.938 when using a simple list of 5 items. Similarly, the calculator by Kim et al [15] designed for a South Korean population reached an AUC of 0.887 and focused on epidemiologic factors over serum markers.

### Limitations

Our study’s main limitation is that the nature of the included articles did not allow for the evaluation of bias as per the Cochrane manual. However, we find that the potential risk for bias is low as each author describes the specific way the calculators are calibrated. Its main strength is that it provides a comprehensive description of available risk calculators and how they can be successfully adapted for different target populations.

### Conclusion

Although most existing risk calculators for prostate cancer were developed with European or North American populations, their calibration for populations in different settings leads to equally high predictive capacities and yields tools that could be used in resource-limited settings. Risk calculators that included multiple items should be used over prior techniques using markers alone in order to decrease unnecessary procedures in healthy populations at lower risk for prostate cancer. Although screening for prostate cancer remains a shared decision based on individual preference and apparent risk, the development and improvement of predictive tools could lead to optimal algorithms that consider patients’ greatest benefit and help for better allocation of health care resources.

## Abbreviations

AUC: area under the curve
CPCC: Chinese Prostate Cancer Consortium
DRE: digital rectal examination
ERSPC: European Randomized Study on Prostate Cancer
MRI: magnetic resonance imaging
PCPT: Prostate Cancer Prevention Trial
PHI: Prostate Health Index
PSA: prostate specific antigen
RC: risk calculator
